# Biomarkers of neutrophil extracellular traps (NETs) and nitric oxide-(NO)-dependent oxidative stress in women who miscarried

**DOI:** 10.1038/s41598-020-70106-x

**Published:** 2020-08-04

**Authors:** Wioleta Justyna Omeljaniuk, Ewa Jabłońska, Marzena Garley, Anna Pryczynicz, Wioletta Ratajczak-Wrona, Katarzyna Socha, Maria Halina Borawska, Angelika Edyta Charkiewicz

**Affiliations:** 1 Department of Pharmaceutical Biochemistry, Faculty of Pharmacy with the Division of Laboratory Medicine, Medical University of Bialystok, Mickiewicza Street 2A, 15-089 Białystok, Poland; 2 Department of Immunology, Faculty of Pharmacy with the Division of Laboratory Medicine, Medical University of Bialystok, Waszyngtona Street 15A, 15-269 Białystok, Poland; 3 Department of Pathomorfology, Faculty of Health Sciences, Medical University of Bialystok, Waszyngtona Street 13, 15-269 Białystok, Poland; 4 Department of Bromatology, Faculty of Pharmacy with the Division of Laboratory Medicine, Medical University of Białystok, Mickiewicza Street 2D, 15-222 Bialystok, Poland; 5 Department of Public Health, Faculty of Health Sciences, Medical University of Bialystok, Szpitalna Street 37, 15-295 Białystok, Poland

**Keywords:** Immunology, Biomarkers, Medical research

## Abstract

Pregnancy loss is a multidisciplinary problem which concerns researchers from the fields of medicine, epidemiology, psychology, and public health. The primary objective of the present study was to explain the potential role of neutrophil extracellular traps (NETs) in the process of spontaneous miscarriage. Enzyme-linked immunosorbent assay to assess the levels of biomarkers of NETs in the serum of examined women was conducted. Furthermore, levels of nitric oxide (NO) and late markers of its action were measured in serum samples. Analyses results demonstrated the existence of NETs in the placental tissue of women who miscarried as well as a simultaneous increase in the levels of myeloperoxidase and pentraxin 3. This clearly confirms the participation of NETs in the course of pregnancy loss. Women who have had a miscarriage but did not show the presence of NETs in their placenta exhibited the highest contents of NO, nitrotyrosine, and malondialdehyde suggesting a different pathway leading to pregnancy loss associated with disturbed oxidative–antioxidative processes. Although study results demonstrate new aspects associated with the formation of NETs they are not, however, sufficient to unambiguously determine the role of NETs in the course of miscarriage.

## Introduction

The term “miscarriage” (Lat. *abortus spontaneus*) defines the spontaneous expulsion of the fertilized ovum from the uterine cavity, in whole or in part, before the fetus is capable of surviving independently^1^. In general, there are two types of miscarriages: fertilized ovum-dependent and mother-dependent. Mother immune-related causes of pregnancy complications account for about 5–10% of all lost pregnancies and doctors and scientists are more interested in uncovering the mechanisms involved in miscarriage^1–6^.

Numerous studies dealing with the subject of miscarriage have suggested a significant role of the state of the oxidoreductive balance between pro-oxidant factors (e.g., free radicals) and the efficiency of the antioxidative systems in the maintenance and normal development of gestation^4–7^. Oxidative stress is a disturbance caused by the imbalance in the production of reactive oxygen species (ROS) and the generation of antioxidants by the organism’s adaptive defense mechanisms^3,8^. ROS are generated by, among others, neutrophils, through the activity of NADPH oxidase, and lead to the formation of oxygen radicals (respiratory burst) causing the system to initiate an antibacterial defensive mechanism^8,9^. Pro-oxidants may be also formed as a result of nitric oxide synthase (NOS) activity in neutrophils responsible for the synthesis of nitric oxide (NO)^10,11^. NO is involved in the nitration of tyrosine’s phenolic units in tissues and blood proteins. Nitrotyrosine (NT), in turn, is responsible for the loss of biological activity of proteins containing tyrosine within their polypeptide chains which causes pathological changes. It has been suggested that NT might be utilized as a marker for the determination of in vivo action of NO. Due to its long half-life, NT is a better indicator of elevated production of NO than that of its metabolites. Elevated levels of NO in serum may have a direct impact on the essential components of the cellular system. NO plays a significant role in the process of lipid peroxidation^9,12,13^. One of the many compounds produced through this process is malondialdehyde (MDA) which has been detected in the mitochondrial fraction of placental cells which, in turn, may lead to complications such as spontaneous and/or recurrent miscarriage^4^.

In 2004, a previously unknown antipathogenic neutrophilic mechanism, later called the neutrophil extracellular traps (NETs), the course of which includes NADPH oxidase, was described. NETs are made up of DNA strands, granule proteins, and histones responsible for killing pathogens and is a consequence of ROS presence in the extracellular space. To the best of our knowledge, no study conducted thus far has focused on the biological role of NETs in miscarriage. Research focusing on a group of women with preeclampsia and recurrent miscarriages has demonstrated that bacterial infections caused by *Brucella abortis* or *Listeria monocytogenes* lead to the recruitment and activation of neutrophils. It was determined that listeriosis in women is directly related to the incidence of miscarriage^11–14^. Other studies showed significantly higher amounts of NETs in the interstitial space of the placenta of women with preeclampsia than in those with normal gestations^12,13^. It has not been, however, determined whether NETs may contribute to pregnancy loss.

The primary objective of the present study was to, therefore, elucidate the role of NETs in the process of miscarriage. Extracellular myeloperoxidase (MPO) and H2A, H2B, and H3 histone expression in fragments of the placenta of women who have miscarried were determined. The concentrations of cell-free DNA (cfDNA) and MPO, both indirect markers of NETs, were assessed in the serum of women who have had a miscarriage. Levels of peptidylarginine deiminase (PADI) 4 which participates in the formation of NETs and of pentraxin 3 (PTX-3), a protective component of the traps as well as the level of NO released into the extracellular space along with the traps and its markers, NT and MDA, were measured. Simultaneous control tests on women who had a normal, successful pregnancy were also conducted.

## Materials and methods

### Experimental and control group

The study group included women aged between 18 and 44 who have experienced early pregnancy loss prior to the 9th week of gestation (n = 84), (Table 1). The women were hospitalized at the Department of Perinatology of the University Clinical Hospital in Bialystok, Poland and at the Division of Obstetrics and Pregnancy Pathology of the Jędrzej Śniadecki District Hospital in Bialystok, Poland. The presence of other diseases such as antiphospholipid antibody syndrome and vein thrombosis was excluded. The control group consisted of women (n = 15) aged between 18 and 32 years old with a normal course of gestation who have delivered a healthy baby. Control group participants were carefully selected and all women who suffered from chronic or temporary diseases during the course of their gestation were excluded.

**Table 1:** Characteristics of examined women.

Characteristic	Control group n = 15	Women “NETs-negative group” n = 51	Women “NETs-positive group” n = 33
**Age (years)**			
Mean ± SE	27.2 ± 0.91	30.8 ± 0.87	29.09 ± 1.09
Median (range)	28 (18–32)	31 (18–44)	29 (18–43)
**Weight (kg)**			
Mean ± SE	61.33 ± 2.19	63.63 ± 1.46	60.41 ± 1.64
Median (range)	57 (53–80)	63 (46–88)	60.5 (43–81)
**BMI (kg/m**^**2**^**)**			
Mean ± SE	22.74 ± 0.81	22.49 ± 0.51	22.03 ± 0.57
Median (range)	21.1 (19.7–30.4)	23 (18.4–34)	21.2 (17.4–31.6)
**Number of children born (before the test)**			
Mean ± SE	0.6 ± 0.23	0.96 ± 0.15	0.91 ± 0.23
Median (range)	0 (0–3)	1 (0–4)	0.5 (0–6)
**Number of previous miscarriages**			
Mean ± SE	0.27 ± 0.15	1.45 ± 0.09	1.19 ± 0.1
Median (range)	0 (0–2)	1 (1–3)	1 (1–3)
**Week of pregnancy miscarriage**			
Mean ± SE	9.4 ± 0.64	8.94 ± 0.34	9 ± 0.51
Median (range)	9 (6–14)	9 (4–16)	9 (4–19)
Number of women smoking cigarettes	1	2	2

### Study material

The experimental material consisted of whole blood serum and placental tissue fragments. Whole blood was collected from women from the study group directly after their miscarriage, whereas, with respect to the control group, it was collected during the first trimester of physiological pregnancy. Placental fragments were collected after the miscarriage or after childbirth. Informed consent was obtained from all participants.

Samples were collected as part of the implementation of a project entitled “Influence of diet and tobacco smoking on the content of zinc, selenium, copper, manganese, and antioxidative status in women with miscarriage”. This study was implemented as part of a supervised research grant of the Ministry of Science and Higher Education (application number: N N405 625538) completed in September of 2011. Study materials were stored at − 80 °C in line with the principles of Good Laboratory Practice. Approval of the Bioethics Committee UMB no. R-I-002/361/2017 was obtained for the extension of the testing panel. A statement to confirm that all methods were carried out in accordance with relevant guidelines and regulations (Declaration of Helsinki).

### Immunohistochemical analysis

In order to detect neutrophils staining with the anti-MPO antibody was performed. Nuclear chromatin was imaged using anti-H2A, anti-H2B, and anti-H3 antibodies.

Tissue containing paraffin blocks were cut using a microtome into 4 μm thick sections and placed on silanized slides. The sections were deparaffinized with xylene and hydrated through a series of alcohol washes. Next, the sections were placed in citrate buffer (pH = 6.0) and incubated in an aqueous bath for 20 min at 98.5 °C to reveal the antigen and were subsequently incubated for 20 min at room temperature. Following that, the sections were incubated with 3% hydrogen peroxide to block endogenous peroxidase and with 1% bovine serum to block nonspecific bonds. In the next stage the slides were incubated with specific antibodies—anti-H2A (Sigma Aldrich, SAB4501371, dilution 1:100), anti-H2B (Sigma Aldrich, SAB4502231, dilution 1:100), anti-H3 (Sigma Aldrich, SAB4500352, dilution 1:200), and anti-MPO (Sigma Aldrich, HPA061464, dilution 1:200) for 30 min at room temperature. After a reaction induced through the use of a polymeric technique (ImmPRESS, Vector Laboratories) the antigen–antibody complex was exposed by incubation with chromogen 3.3′-diaminobenzidine (DAB, Vector Laboratories). The cellular nuclei were stained with hematoxylin. The evaluation of immunohistochemical staining was performed with a light microscope using 400× magnification for women with miscarriages, groups A and B, and a magnification of 200× for the control group (A). NETs or neutrophils were counted in the intervillous space and classified as absent or present.

### Indirect method of NO assessment based on Griess reagent

NO levels in serum were determined spectrophotometrically based on the cadmium reduction of NO_3_^−^ to NO_2_^−^. Serum samples (100 μL) were mixed with equal volumes (100 μL) of Griess reagent (Sigma-Aldrich) in a 96-well plate. Absorbance was measured at 540 nm in a microplate reader (UVN-340 ASYS Hitech GmbH microplate reader; Biogenet, Eugendorf, Austria). The micromolar concentration of nitrite was calculated using a standard curve prepared using sodium nitrite (0.1 M).

### Immunoenzymatic methods

The level of MPO in serum samples was measured using the Human Myeloperoxidase Quantikine ELISA Kit (R&D Systems). The test's minimum detection range was 0.2 ng/mL with a sensitivity of 0.062 ng/mL. NT was quantified using Nitrotyrosin ELISA (Immundiagnostik AG) with a minimum range detection of 264 nM and a limit of blank of 0.853 nM. MDA was estimated using the Human Malondialdehyde (MDA) ELISA Kit, Competitive ELISA, (MyBioSource). The sensitivity of the kit was 1.0 ng/mL with a minimum detection range of 94–102%. PADI 4 was determined through the utilization of a Human Protein-Arginine Deiminase type-4 (PADI4) ELISA Kit (MyBioSource). The minimum detection range for samples tested was 0.156 ng/mL and test sensitivity was < 0.039 ng/mL. PTX-3 was estimated using the Human Pentraxin 3 SimpleStep ELISA Kit (Abcam) with a minimum detectable dose of 3.4 pg/mL and a range of 15.6–1,000 pg/mL. The amount of circulating cfDNA in the blood serum was determined using the immunofluorescent Circulating DNA Quantification Kit (Abcam). This kit provides a rapid (30 min/1 sample), convenient method for the assessment of cfDNA. Serum was treatment with a DNA digestion buffer and DNA recovery was performed through the utilization of a Fast-Spin Column. DNA was then fluorescently quantified without interference. Fluorescence was obtained only from purified cfDNA. The linear detection range was 0.1–100 ng/μL with a sensitivity ≥ 0.1 ng/μL. DNASE1 level in serum samples was assessed using the Enzyme-linked immunosorbent Assay Kit For Deoxyribonuclease I (Cloud-Clone Corp.) with a detection range of 78–5,000 pg/mL and a minimum detectable dose of DNASE1 being < 30 pg/mL.

### Statistical analysis

Statistical analysis was conducted using the Statistica, version 13.3, program (StatSoft, Inc., Tulsa). All data is reported as the mean ± SD (standard deviation) (Table 3) or mean ± SE (standard error), median and range (Table 1). Normality of data distribution was tested through the employment of a Shapiro–Wilk test. Two study-considered variables were analyzed using a Student’s *t*-test for parametric distribution and the Mann–Whitney *U* test for nonparametric distribution. Correlations were performed using the Spearman correlation coefficient. *P*-values of less than 0.05 were considered statistically significant.

### Statement of ethics

The authors have no ethical conflicts to declare as this is an original article.

## Results

Data analysis allowed us to distinguish 2 study groups of women: the first group consisted of women who miscarried and did not have NETs in their placental tissue specimen—this group is referred to as the “NETs-negative group” and the second group included women who suffered a miscarriage but had NETs in their placental tissue specimen—this group is referred to as the “NETs-positive group”.

Table 1 shows data obtained through a questionnaire filled out by the women examined in this study. There were no statistically significant differences between the parameters presented in the table for both examined groups of women.

### Assessment of MPO and histone expression in the placenta

**Figure 1:**
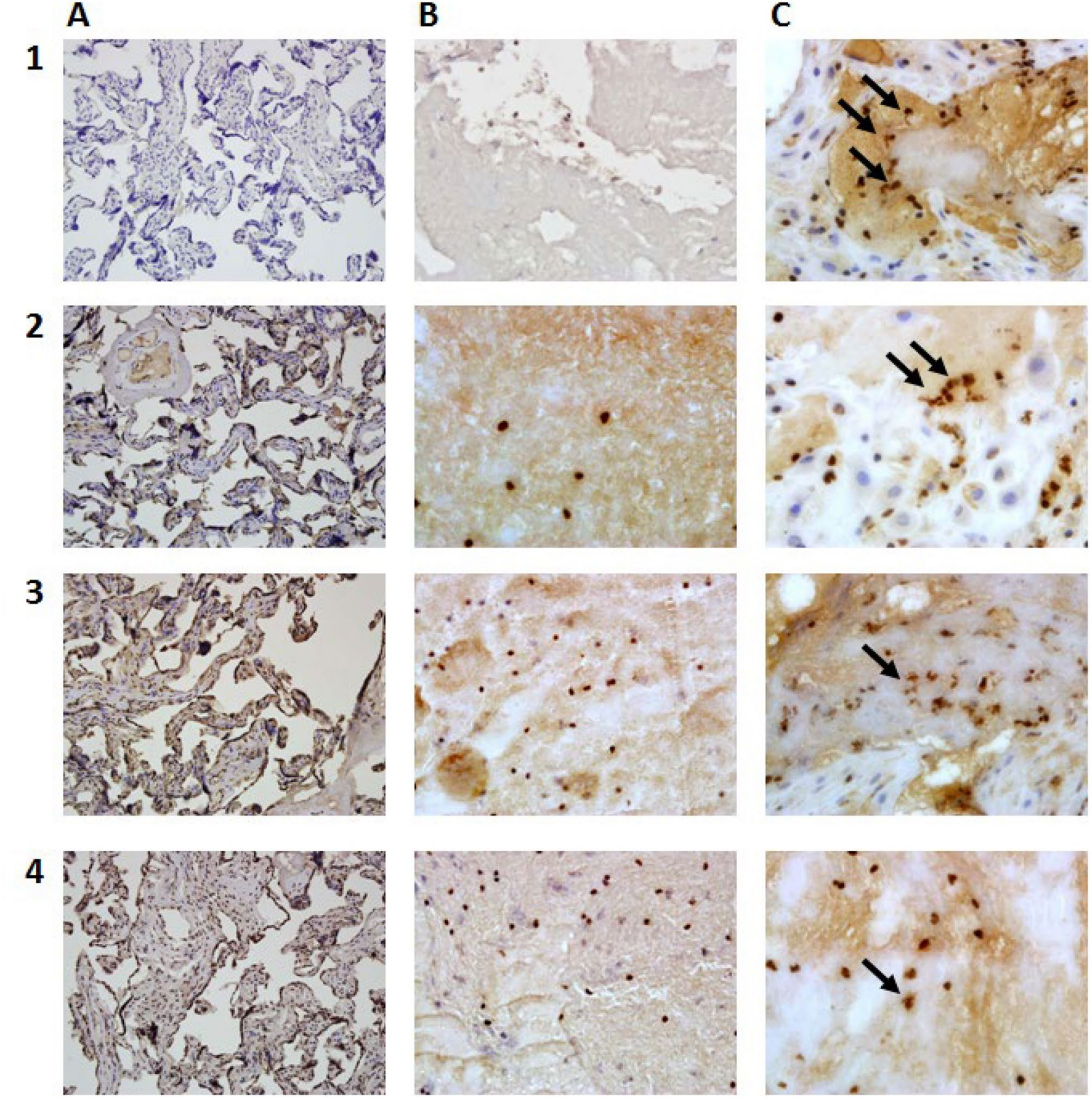
The presence of NETs (MPO and histones: H2A, H2B, and H3) in the chorion and the placental decidua. Chromogen 3.3′-diaminobenzidine: (1) anti-MPO, (2) anti-H2A, (3) anti-H2B, (4) anti-H3 antibodies; (**A**) control group, without granulocytes (magnification 200x); (**B**) women with miscarriage, with granulocytes (magnification 400x); (**C**) women with miscarriage, with NETs (arrows) (magnification 400x).

The presence of NETs (MPO and histones: H2A, H2B, and H3) was assessed in the chorion and the placental decidua (Fig. 1).

No NETs could be found in the placental tissue of the control group.

The absence of NETs in the placental tissue was found for some of the women who have had a miscarriage providing a basis for dividing the study group into two separate groups with these women classified into the NETs-negative group. All remaining women who had suffered a miscarriage and whose placental tissue showed the presence of NETs markers: MPO, H2A, H2B, and H3 histones were included in the NETs-positive group.

### Analysis of NO levels

Analysis of NO in the serum of all examined groups demonstrated significantly higher levels of total NO in the samples of the NETs-negative group than those of the control group and the NETs-positive group with results showing statistically significant lower levels of total NO in women from the NETs-positive group than those of women from the control group (Table 2).

**Table 2:** Parameters related to the formation and release of NETs determined in the blood serum of examined women.

The examined women	Assessed parameters Mean ± SD							
	NO [μM]	MPO [ng/mL]	NT [nM]	MDA [ng/mL]	PADI 4 [ng/mL]	PTX-3 [pg/mL]	cfDNA [ng/1μL]	DNASE1 [pg/mL]
Control group n = 15	53.293 ± 14.335	123.121 ± 26.362	346.583 ± 48.190	6.712 ± 0.561	9.472 ± 1.045	20.908 ± 3.629	1.128 ± 0.191	927.205 ± 159.861
Women “NETs-negative group” n = 51	75.846* ± 15.559	215.899* ± 54.409	574.623* ± 68.409	7.453 ± 0.717	7.798* ± 1.138	31.419* ± 3.918	0.409* ± 0.093	1,300.366* ± 322.052
Women “NETs-positive group” n = 33	39.325*^#^ ± 8.573	512.447*^#^ ± 66.503	406.366^#^ ± 66.988	6.695^#^ ± 0.744	8.799^#^ ± 0.874	54.680*^#^ ± 8.953	0.365* ± 0.031	1779.067*^#^ ± 463.066

*Statistically significant difference with control (*p* ≤ 0.03); ^#^statistically significant difference between women of "NETs-negative group" and women of "NETs-positive group" (*p* ≤ 0.01).

### Analysis of MPO levels

According to the present study's results women who had a miscarriage showed higher levels of MPO than those who did not. Women classified into the NETs-positive group showed the highest mean concentration of MPO which was over twofold higher than that of women included in the NETs-negative group and fourfold higher than those of the control group (Table 2).

### Analysis of NT levels

Analysis of NT levels within the samples of all women included in the study demonstrated significantly higher levels of NT in serum samples of participants from the NETs-negative group than in those from the control group and the NETs-positive group (Table 2).

### Analysis of MDA levels

Analysis of MDA levels contained in serum samples did not reveal any statistically significant differences between women who had had a miscarriage and women who did not. However, women classified into the NETs-positive group showed lower levels of MDA than those from the NETs-negative group (Table 2).

### Estimation of PADI 4 levels

According to the obtained results, women who miscarried showed reduced levels of PADI 4 in comparison to women from the control group who did not. The lowest level was observed for patients who fell into the NETs-negative group which was statistically significant when compared with those from the control group and the NETs-positive patients (Table 2).

### Determination of PTX-3 levels

Our results demonstrated a statistically significant increase in the levels of PTX-3 in women who had a miscarriage when compared with women of the control group. The highest levels of PTX-3 were seen for patients categorized as NETs-positive and were significantly higher than those of NETs-negative group patients (Table 2).

### cfDNA concentration assay

Results of cfDNA analysis demonstrated significantly lower concentrations of this factor in samples of women from both the NETs-positive and NETs-negative groups than in those of women from the control group (Table 2).

### DNASE1 level assessment

Measurement results of DNASE1 levels showed significantly higher levels of DNASE1 in samples of women who have had a miscarriage in relation to those of the control group participants. Additionally, higher DNASE1 concentrations in NETs-positive patients' samples compared to those falling into the NETs-negative group were observed (Table 2).

### Correlations

Table 3 shows the results of a correlation analysis performed on the basis of the assessed parameters.

**Table 3:** Correlations between assessed parameters.

The examined women	Correlated parameters	Correlation coefficient
Control group	MPO versus cfDNA	r = 0.547 (*p* = 0.028)
	NT versus PADI 4	r = − 0.576 (*p* = 0.02)
	MDA versus NT	r = 0.522 (*p* = 0.038)
Women “NETs-negative group”	MPO versus NT	r = 0.364 (*p* = 0.008)
	MDA versus NT	r = 0.328 (*p* = 0.018)
Women “NETs-positive group”	MPO versus PADI 4	r = 0.374 (*p* = 0.029)

## Discussion

The presence of basic structural elements of NETs in the placental tissue and their high levels in the serum of women who miscarried clearly indicates the participation of NETs in the premature termination of pregnancy. Similar results were obtained by Gupta et al., who showed the relationship between preeclampsia, which poses a threat to the life of the fetus and the mother through excessive neutrophil activation, and the number of NETs^13,15,16^. Giaglis et al. confirmed earlier discoveries indicating that pregnancy is associated with inflammation characterized by neutrophil activation^17,18^. Their study provides evidence that during gestation neutrophils release excessive NETs and that their release is regulated by the granulocyte colony-stimulating factor (G-CSF) and/or sex hormones. Disturbance in the hormonal balance results in an elevated production of NETs which is associated with damage to the local tissues and complications of preeclampsia and even pregnancy loss^17^. Other studies showed that interleukin (IL)-8 of placental origin strongly activates neutrophils to form NETs^13^. It has also been proven that tissue factor, a component of NETs, is a primary contributor in the process of miscarriage through the induction of a cascade of ROS/NETs reactions^13^.

The preceding research could not prove a direct relationship between the formation of NETs and pregnancy loss. Although scarce, literature points to a relation between NETs and the presence of auto-antibodies^12,19^. Hahn et al. established a positive correlation between antiphospholipid antibodies in the blood of women who miscarried and the oxidative damage of the placental tissue through the generation of ROS in activated neutrophils^12,19,20^. Excessive production of and/or prolonged presence of NETs leads to the formation of antinuclear cytoplasmic antibodies (ANCA) and antibodies which act against the body's own DNA which forms the structure of NETs.

Contrary to the results of studies that have reported high levels of cfDNA during the course of various diseases with the participation of neutrophils, in this study low levels of cfDNA in the serum of women who had a miscarriage were obtained. For this reason, it is difficult to compare the results of our study with those of other studies considering the fact that both the control group and the study group were comprised of pregnant women whose majority of parameters deviate from reference values. It was surprising to observe negative results in relation to NETs in the placenta and low cfDNA levels in serum of some women who miscarried with simultaneously elevated other parameters associated with the production of NETs. The present work's results suggest the formation of NETs in locations other than the placenta as well as, perhaps, their inefficient degradation at the early stages of pregnancy loss. High DNASE1 concentrations demonstrated within this research determining the degradation of NETs, might be the cause of low levels of cfDNA in serum. On the other hand, low serum concentrations of cfDNA may also be the result of DNA binding in the NETs, because, despite high DNASE1 concentrations, it may be inactive in the normal degradation of NETs over time^21^. Confirmation of this study's conclusion that low levels of cfDNA and placental dysfunction of pregnant women can also be found in research completed by Gerson et al.^22^. It is necessary to continue research in the field of measuring DNase activity, through, for example, the application of the SRED (single radial enzyme diffusion) method and cfDNA quantification in EDTA (makes DNase inactive) plasma.

In this study, the observed positive correlation between the levels of cfDNA and MPO in the control group is similar to the results of earlier research involving a group of healthy individuals which suggested the use of these factors as intermediate markers of released NETs. High levels of MPO in both of the present study's patient groups indicate its significant role in miscarriages involving NETs. Metzler et al. demonstrated that neutrophils of people with complete MPO deficiency do not produce NETs and pointed out the fact that the formation of NETs requires high local levels of MPO^23^.

Earlier research on PTX-3 in NETs showed that PTX-3 plays a key role in regulating the proper development of pregnancy and the inflammatory response, such as in preeclampsia. An uncontrolled inflammatory reaction is believed to be the primary cause of unexplained recurrent pregnancy loss^24,25^. It has been proven that the gene responsible for PTX-3 activation is activated during the early gestation period. PTX-3 is expressed in endometrial stromal cells and trophoblast influences its expression in the swollen uterine mucosa during implantation. Ibrahim et al. compared levels of PTX-3 in the serum of patients with primary and recurrent pregnancy loss and in women with successful pregnancies during the same week of pregnancy. Their results showed that levels of PTX-3 were highest in women who miscarried and correlated with the number of earlier pregnancy losses^25^. The results of this study correspond with those of Ibrahim et al. Furthermore, within this study, the highest values of PTX-3 were seen in samples obtained from women falling into the NETs-positive group which indicates that levels of PTX-3 increase along with an increase in the associated NETs factors. According to our results, among women who had a miscarriage, only two parameters showed significant increases with their highest values associated with the presence of NETs in the placenta. Increasing concentrations of the proinflammatory MPO and the protective PTX-3 having a high likelihood of being associated with NETs formation can be considered to be early miscarriage markers,. It is hypothesized that PTX-3 plays a regulatory role in the formation of NETs through, among others, binding histones and alleviating the cytotoxic effect on endothelial cells.

A mouse model utilized by Erpenbeck et al. was a source of a discovery associated with the regulation of NETs formation and fetal loss due to the accumulation of activated neutrophils in the placenta. The authors demonstrated a significant contribution of PADI 4 in the formation of NETs during pregnancy. The deficiency of PADI 4 correlates with the reduced production of NETs which, in turn, reduces inflammation and eventually protects against fetal loss^26^. Within the present study, the positive correlation between PADI 4 and MPO observed among NETs-positive women confirms the participation of PADI 4 in the formation of NETs. A minor decrease in the levels of PADI 4 in women who had a miscarriage is surprising and suggests the existence of a regulatory mechanism that reduces the formation of NETs during pregnancy.

The negative correlation between PADI 4 and NT, which correlated positively with MDA, recorded in the control group shows that pro-oxidative processes are intensified in pregnancy through a different route than ROS/NETs. During a miscarriage oxidative stress occurs at a systemic level and could be the result of at least four mechanisms: an imbalanced influence of ROS on the metabolism of eicosanoids, the direct embryotoxic effect of ROS at the intracellular level, induction of ROS leading to endothelial dysfunction within the trophoblast, and interactions between the emotional state and oxidative stress^10,21,25^.

Our results showed a rapid increase in the amount of NO and NT in NETs-negative women indicating a set of processes leading to miscarriage when compared to the results of women from the NETs-positive group. During physiological pregnancy NO plays the role of a mediator of vascular homeostasis, modulating both the utero-placental and fetal-placental circulation. Literature shows that during physiological pregnancy, the total amount of nitrites, the final products of NO metabolism, considerably increases in blood circulation returning to the value from before pregnancy 12 weeks after childbirth^27–29^. Low concentrations of NO and changes in the amount of NT (a late marker of NO-dependent oxidative modification of proteins) observed among women in the NETs-positive group indicates a disturbance in the oxidative–antioxidative balance which might be caused due to the excessive increase in the level of free radicals.

In this study, the positive correlation between the levels of MDA and NT in women falling into the NETs-negative group suggests a progression in the processes of oxidative stress and an increase in its effects. There were no significant differences in the levels of MDA in samples of NETs-positive and control group patients which suggests a low intensity of lipid peroxidation processes during the formation of NETs.

Jauniaux et al. claim that in normal pregnancies, the earliest stages of development take place in a low oxygen (O_2_) environment. This physiological hypoxia of the early gestational sac protects the developing fetus against the deleterious and teratogenic effects of O_2_ free radicals (OFRs). In a miscarriage, the development of the placento-decidual interface is severely impaired leading to the early and widespread onset of maternal blood flow and major oxidative degeneration. This mechanism is common to all miscarriages, with the time at which it occurs within the first trimester depending on the etiology^3^. In our research, the absence of statistically significant differences in terms of age, body mass index, tobacco smoking, weight, number of children born, number of earlier miscarriages, and the week of miscarriage excluded these factors as causes of pregnancy loss in the studied groups.

**Figure 2:**
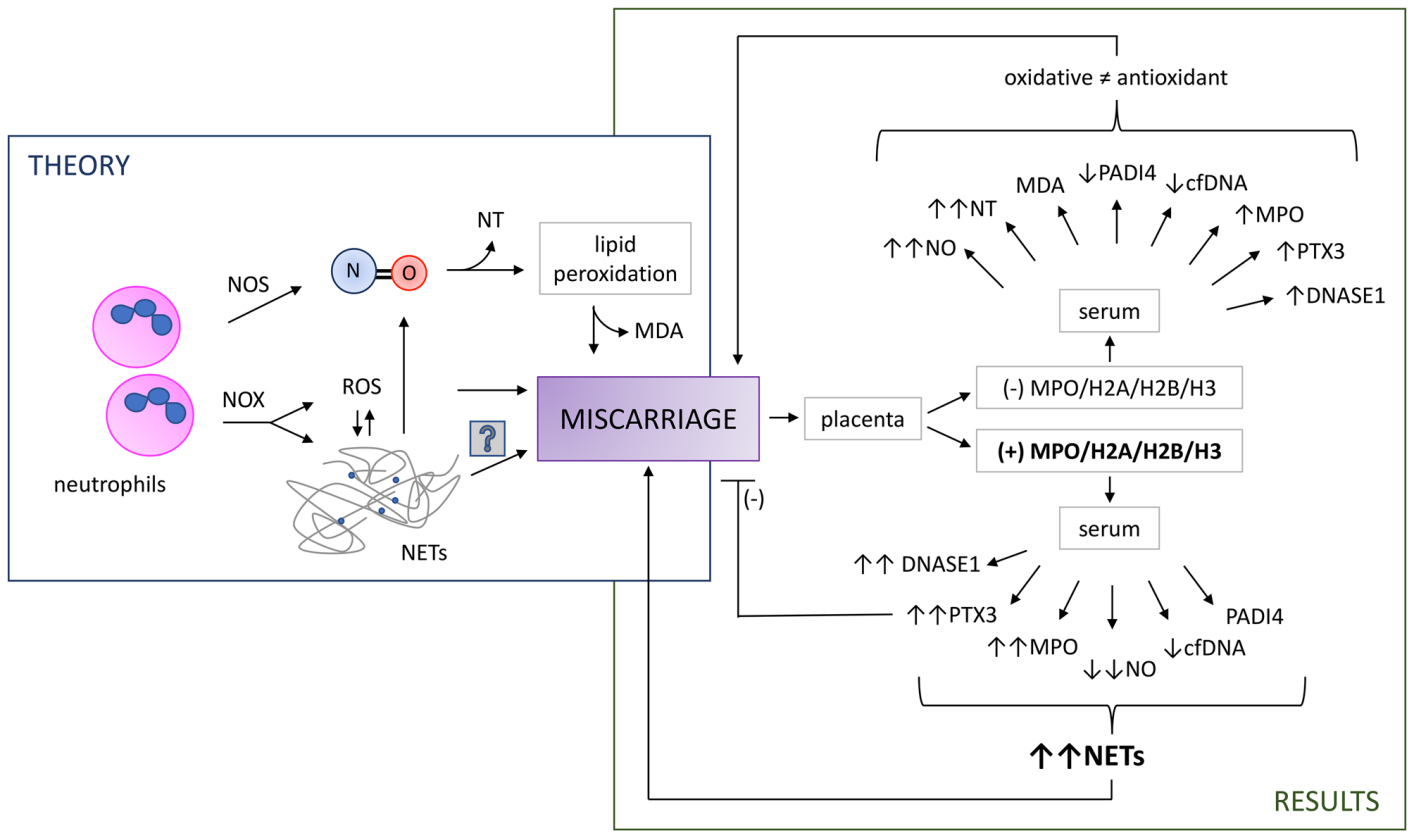
Proposed mechanism of NO/NETs-dependent oxidative stress formation in women who had miscarried. NOS-dependent neutrophils-derived NO promotes miscarriage by lipid peroxidation, by NT and MDA. NOX-dependent stimulating ROS production, could affect miscarriage. We hypothesized if NOX-dependent NETs forming is the cause of miscarriage. Based on observed two population of measured placenta and serum markers we suggest two different NETs-involving mechanisms occurrences of miscarriage. In NETs positive patients (increase MPO and PTX3, decrease NO and cfDNA in serum), miscarriages were supported by direct harmful influence of NETs components. Whereas, in NETs negative patients (increase NO and NT, decrease PADI4 and cfDNA, but increase MPO and PTX3 in serum) with very high-level of oxidative stress markers (NO and NT)—dysregulation of oxidative and antioxidant balance, which suggest oxidative stress-dependent impairment and miscarriage. Abbreviations: NETs, neutrophil extracellular traps; NOS, nitric oxide synthase; NOX, NADPH oxidase; ROS, reactive oxygen species; NT, nitrotyrosine; MDA, malondialdehyde; PADI4, peptidylarginine deiminase 4; cfDNA, cyrkulating free DNA; MPO, myeloperoxidase; PTX-3, pentraxin 3; histones 2A, 2B, 3—H2A, H2B, H3.

Based on the results of our own research and the available scientific knowledge, we proposed a probable mechanism of NO/NETs-dependent oxidative stress formation in the tissue of women who have miscarried is presented in Fig. 2. Factors responsible for the induction of the first miscarriage might also be factors responsible for subsequent pregnancy loss. A multidisciplinary study involving genetic, hormonal and anatomical research might be able to explain the majority of the reasons behind miscarriage but may omit causes that seem to be closely linked to immune dysfunctions^30,31^. It is, therefore, important that research focuses on such immune-related causes of miscarriage. The results of this study allowed the identification of two groups of women who miscarried due to placental changes. Interpretation of these results shows the possibility of two, slightly different, mechanisms of pregnancy loss associated with the formation of NETs and a disturbance in the oxidative–antioxidative balance. Results obtained for the NETs-positive group provide unquestionable evidence showing the participation of NETs in the course of miscarriage. Further research should be conducted in order to elucidate the actual role of the formation of NETs during pregnancy loss. Several new aspects associated with the formation of NETs have been explained in this study however, a fuller understanding of this phenomenon might enable the future prevention of pregnancy loss through the modulation of NETs formation and/or destruction.
